# *Magadhaideus*, a new genus of the tribe Plectoderini with the description of a new species from China (Hemiptera, Fulgoromorpha, Achilidae)

**DOI:** 10.3897/zookeys.724.14742

**Published:** 2017-12-21

**Authors:** Jian-Kun Long, Zhi-Min Chang, Lin Yang, Xiang-Sheng Chen

**Affiliations:** 1 College of Animal Sciences, Guizhou University, Guiyang, Guizhou, 550025, P. R. China; 2 Institute of Entomology, Special Key Laboratory for Development and Utilization of Insect Resources of Guizhou, Guizhou University, Guiyang, Guizhou, 550025, P. R. China

**Keywords:** Achilid, distribution, Fulgoroidea, new taxa, planthopper

## Abstract

A new planthopper genus and species from China, *Magadhaideus xiphos* Long & Chen, **gen. et sp. n.** (Hemiptera: Fulgoromorpha: Achilidae: Plectoderini), is described and illustrated. A new combination, *Magadhaideus cervina* (Fennah, 1956), **comb. n.** transferred from *Magadha* Distant and a key to species of the new genus are also given.

## Introduction

The planthopper tribe Plectoderini (Hemiptera: Achilidae) established by Fennah (Fennah, 1950), containing about 98 genera 335 species to date (Bourgoin 2017), is the largest tribe of Achilidae (Hemiptera: Fulgoromorpha). It is also the most widely dispersed in the world.

Approximately four tribes, 20 genera, and 99 species of Achilidae are known in China. Plectoderini consists of 15 genera and 79 species. Almost all members of the tribe in China are distributed in the Oriental region, especially in southern China. Here, a new genus and species of the tribe from South China are described and illustrated. A new combination and a key to the species of the new genus are also provided.

## Materials and methods

The morphological terminology and measurements used in this study mainly follow Chen et al. (1989) and Yang and Chang (2000). The standard terminology for hind and forewing venation follow Bourgoin et al. (2015). The methods follow Long et al. (2014). The genital segments of the examined specimens were macerated in 10% KOH and drawn from preparations in glycerine jelly using an Leica M125 stereomicroscope. The type material is deposited in the Institute of Entomology, Guizhou University, Guiyang, China (GUGC).

## Taxonomy

### Genus Magadhaideus

Taxon classificationAnimaliaORDOFAMILIA

Long & Chen gen. n.

http://zoobank.org/C6B86A2A-6A05-4022-B8F4-FC21356E323F

Figs 1–4 , 5–15

#### Type species.

*Magadhaideus xiphos* Long & Chen, sp. n., here designated.

#### Differential diagnosis.

The new genus and *Magadha* are readily distinguished from other known genera in the tribe Plectoderini by mesonotum with a transverse callus on anterior third of disc (Fig. 5). The new genus differs from *Magadha* in: pygofer (Fig. 11) in lateral view with dorsal margin distinctly shorter than ventral margin (dorsal margin at least as long as ventral margin in *Magadha*); medioventral process (Fig. 12) broad and short, with a small sharp process lateroapically (relatively slender and without small sharp process lateroapically in *Magadha*); genital style (Fig. 13) without a finger-like process from near base of dorsal margin (with a finger-like process from near base of dorsal margin in *Magadha*); phallobase (Figs 14–15) with apical half branched into much more and longer processes (apical half, in *Magadha*, at most branched into one dorsal, one ventral and two lateral lobes, all of them short).

**Figures 1–4.**
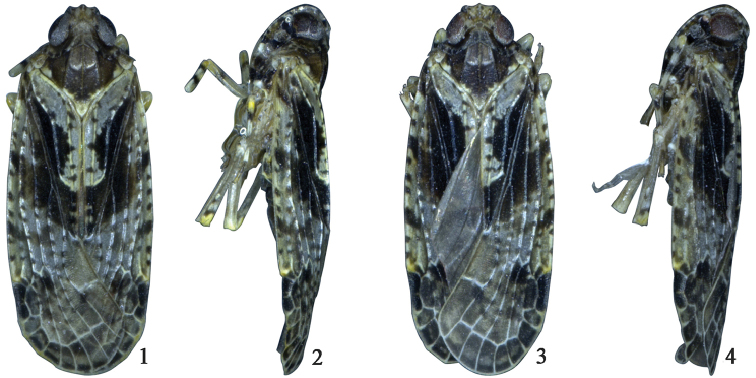
*Magadhaideus xiphos* sp. n. **1–2** Male habitus (dorsal and lateral views) **3–4** Female habitus (dorsal and lateral views).

**Figures 5–15.**
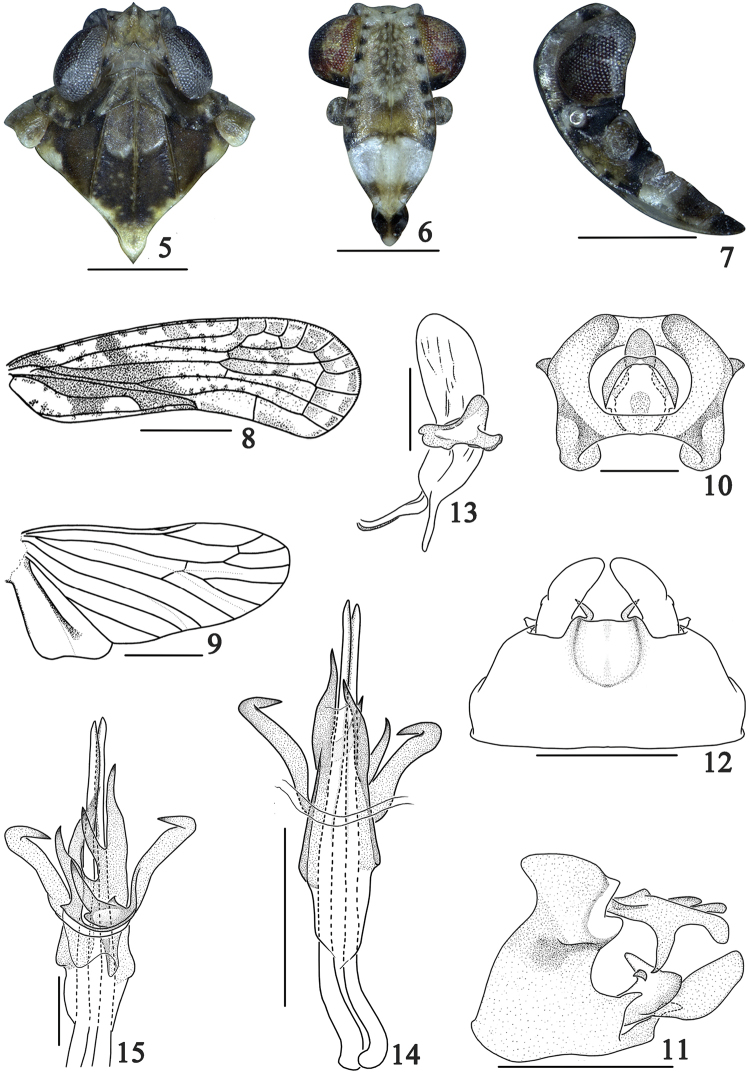
*Magadhaideus xiphos* sp. n. **5** Head and thorax, dorsal view **6** Face **7** Head, lateral view **8** Forewing **9** Hindwing **10** Anal segment of male, dorsal view **11** Male genitalia, lateral view **12** Male genitalia, ventral view **13** Left genital style, dorsal view **14** Aedeagus, dorsal view **15** Aedeagus, ventral view. Scale bars: 0.3 mm (**10, 13, 15**); 0.5 mm (**5–7, 11–12, 14**); 1 mm (**8–9**).

#### Description.

Width of head with eyes 0.8 times wider than width of pronotum. Vertex (Figs 1, 3, 5) with disc distinctly depressed, width at base wider than length in midline, median carina obsolete, anterior margin carinate, angularly convex, lateral margins slightly foliate, diverging basally, posterior margin subangularly concave. Triangular areolets (Fig. 5) at lateroapical angles of head distinct. Frons (Fig. 6) slightly convex in lateral view, with length in midline distinctly longer than maximum width, median carina complete , lateral margins slightly foliate, straight diverging to below level of antennae then gradually incurved to suture. Clypeus (Fig. 6) with median and lateral carinae distinct, length in midline shorter than frons. Rostrum reaching base of hind femurs, with length of subapical segment shorter than apical segment. Antenna (Figs 2, 4, 6–7) subglobose, not sunk in a depression. Ocelli (Figs 2, 4, 7) separated from eyes. Eyes (Figs 2, 4, 7) almost not excavate beneath. Pronotum (Figs 1, 3, 5) with length in midline as long as length behind eyes, with anterior margin roundly convex, posterior margin subangularly excavate about 130 degrees; median carina distinct, lateral carinae straight, posteriorly diverging and reaching hind margin, with length 1.5 times to length of median carina; lateral lobe slightly inclined antero-ventrally. Mesonotum (Figs 1, 3, 5) with three carinae distinctly, length in midline longer than vertex and pronotum combined, area between lateral carinae with one transverse callus at anterior third. Forewing (Figs 1–4, 8) with costal margin slightly convex; apical margin roundly convex; posterior margin angularly excavate (160 degrees) at apex of clavus; vein Cu_1_ forking slightly basally of ScP+R fork, equal to level of veins Pcu and A1 fork, vein MP forking nearly level of nodal line, clavus terminating slightly distally of middle. Hindwing (Fig. 9) Cu with two branches, partly fused with M3+4, CuP and Pcu single, A1 two branched, A2 not reaching wing margin and enlarged apically. Post-tibiae with one lateral spine basalto middle.

#### Male genitalia.

Each side of anal segment (Figs 10–11) with a strong spinous process, directed ventrally. Pygofer (Fig. 11) in lateral view with dorsal margin distinctly shorter than ventral margin, medioventral process (Fig. 12) broad and short, lateroapical margin produced in a small sharp process. Genital style (Fig. 13) without a finger-like process from near the base of dorsal margin, only a larger process rising from near middle of dorsal margin. Aedeagus with phallobase (Figs 14–15) sheath-shaped, asymmetrical, apical half branched into several long processes which narrowing apically and with apexes sharp. Aedeagal appendages (Figs 14–15) relatively straight, clearly exceeding the apical margin of phallobase.

#### Etymology.

The genus name, which is masculine, is a combination of “*Magadha*” (name of the related genus) and “-*ideus*” (similar to), which indicates the new genus is similar to the genus *Magadha*.

#### Host plant.

Unknown.

#### Distribution.

Oriental region (South China).

#### Key to species of *Magadhaideus* Long & Chen, gen. n.

**Table:** 

1	Forewing with a dark brown stripe from base to apex of clavus (Figs 1–4, 8); medioventral process of pygofer with two small lateroapical processes, directed outward, apical margin truncate (Fig. 12); genital style with dorsal process large and broad, almost not branched into lobes (Fig. 13)	***M. xiphos* sp. n.**
–	Forewing without stripe from base to apex of clavus (Fennah 1956); medioventral process of pygofer with two lateroapical processes, directed inward, apical margin not truncate (Fennah 1956: fig. 15: B); genital style with dorsal process distinctly branched into three lobes (Fennah 1956: fig. 15: C)	***M. cervina* comb. n**.

### Magadhaideus cervina

Taxon classificationAnimaliaORDOFAMILIA

(Fennah, 1956) comb. n.

Magadha cervina Fennah, 1956: 488.

#### Material examined.

No specimens of this species were available for this study. But following Fennah, 1956: fig. 15: A–E, the species here is transferred into *Magadhaideus* gen. n.

#### Host plant.

Unknown.

#### Distribution.

China (Sichuan: Emeishan, 29°32'N, 103°19'E).

### Magadhaideus xiphos

Taxon classificationAnimaliaORDOFAMILIA

Long & Chen sp. n.

http://zoobank.org/F5AD67A1-8000-4149-AC31-8C0035C62BBB

Figs 1–4 , 5–15 , 16–20

#### Type material.

Holotype: ♂, CHINA, **Fujian**: Wuyishan National Natural Reserve (26°54'N, 116°42'E), sweeping, 21 August 2013, Y. Liu. Paratypes, **Fujian**: 2 ♂♂, same data as holotype; 2 ♂♂ 2 ♀♀, Wuyishan National Natural Reserve (26°54'N, 116°42'E), sweeping, 21 August 2013, Y.-Y. Liu; 2 ♂♂, Wuyishan National Natural Reserve (26°54'N, 116°42'E), sweeping, 24 August 2013, Y. Liu. **Shanxi**: 1 ♂, Lishan National Natural Reserve (35°25'N, 111°58'E), sweeping, 24 July 2012, P. Zhang. **Jiangxi**: 1 ♂ 2 ♀♀, Jiulianshan National Natural Reserve (24°38'N, 114°33'E), 600–700 m, sweeping, 19–27 July 2009, Z.-H. Meng. **Zhejiang**: 2 ♀♀, Qingliangfeng National Natural Reserve (30°07'N, 118°51'E), sweeping, 25 July 2009, T.-T. He. **Guizhou**: 1 ♀, Maolan National Natural Reserve (25°30'N, 107°98'E), sweeping, 4 August 2006, F.-L. Xu. **Guangdong**: 2 ♀♀, Nankunshan National Natural Reserve (23°38'N, 114°38'E), sweeping, 23 August 2010, Y.-J. Li.

#### Diagnosis.

The salient features of the new species include the following: forewing with a dark brown stripe from base to apex of clavus (Figs 1–4, 8); medioventral process of pygofer with two small lateroapical processes, directed outward, apical margin truncate (Fig. 12); and phallobase of aedeagus with apical 1/2 branched into seven long processes (Figs 14–15).

#### Description.

*Measurements*. Body length (from apex of vertex to tip of forewing): male 4.2–4.6 mm (n = 7), female 4.9–5.1 mm (n = 10); forewing length: male 3.5–3.9 mm (n = 7), female 4.2–4.3 mm (n = 10).

*Colouration*. Head pale yellowish brown. Vertex (Figs 1, 3, 5) along each lateral margin with one dark brown marking at base and another one brown marking at level of anterior margin of eyes; along midline with two brow to dark brown markings apically. Triangular areolets (Figs 1, 3, 5) at lateroapical angles of head with a dark brown marking. Frons (Fig. 6) with seven dark brown markings along lateral margin, disc in middle scattered ivory-white dots between eyes. Postclypeus ivory-white, with a transverse brown band apically. Frontoclypeus (Fig. 6) dark brown, with the base and apex ivory-white. Rostrum yellowish brown, with apex brown. Genae, as in Fig. 7, with four and two transverse short dark brown stripes, respectively along anterior margin and above eyes; another two large parallel transverse stipes, one above and one beneath antennae. Eyes (Figs 1–7) generally reddish brown; ocellus (Figs 2, 4, 7) yellowish white. Antennae (Figs 2, 4, 6–7) yellowish brown. Pronotum (Figs 1, 3, 5) brown, lateral lobe with five dark brown areas along posterior margin. Mesonotum (Figs 1, 3, 5) dark brown, posterior two-thirds between lateral carinae with few scattered ivory-white dots, apical angle and areas along posterior margin between lateral carinae ivory-white, each lateral angle with a large ivory-white marking along posterior margin. Tegula (Figs 1–5) yellowish brown, along posterior margin paler. Forewing (Figs 1–4, 8) greyish white, with a broad irregular longitudinal dark brown band from base to apex of clavus, small variably sized markings scattered as in Fig. 8. Hindwing pale brown, veins brown. Legs (Figs 2, 4) ivory-white to pale yellowish brown; tibiae yellow basally, the first tarsomeres dark brown; pro- and mesofemora with a dorsal dark brown spot near base, pro- and mesotibiae with a ring dark brown spot respectively near base and in the middle; hind tibia with two ring dark brown spot near base. Abdomen dark brown.

*Head and thorax.* Ratio width of vertex at posterior margin to its length in midline 1.8 (Fig. 5), anterior third produced before eyes. Ratio length of frons in midline to its maximum width 1.3, ratio maximum of width to width at apex 1.9. Ratio length of postclypeus in midline to length of frons 0.5 (Fig. 6). Rostrum with ratio apical to subapical segment 1.2. Lateral lobes of pronotum with three short longitudinal carinae behind eye, ratio length in midline to length of vertex 0.8 (Fig. 5). Mesonotum (Fig. 5) in midline 5.1 times longer than pronotum, 2.3 times longer than pronotum and vertex combined. Forewing (Fig. 8) with ratio of length to maximum width 2.9, vein R with subapical cell. Hindwing (Fig. 9) with length to maximum width ratio of 2.0. Post-tibiae with a lateral spine in basal two-fifths, spinal formula 7–6–6.

*Male genitalia*. Anal segment in dorsal view (Fig. 10) with ratio length to maximum width 1.2, basal margin roundly convex in middle, apical margin slightly convex to subtruncate, anal style not exceeding apical margin of anal segment; in lateral view (Fig. 11) apex of anal segment bent ventrally, apical margin roundly convex, lateral margin near middle with a strong spinous process, directed ventrally. Pygofer in lateral view (Fig. 11) with posterior margin strongly sinuate, medioventral process (Fig. 12) short and broad, with two small lateroapical processes, directed outward, apical margin truncate. Genital style (Fig. 13) relatively narrow and long with apical margin roundly convex, a large and broad process, with apical margin sinuate, rising from middle of dorsal margin. Aedeagus (Figs 14–15) asymmetrical, phallobase with apical half branched into seven long processes which narrowing apically, acute at apexes; among them, two lateral processes with apexes bent, directed inwards. Phallic appendages straight, xiphoid, distinctly exceeding apical margin of phallobase.

**Figures 16–20.**
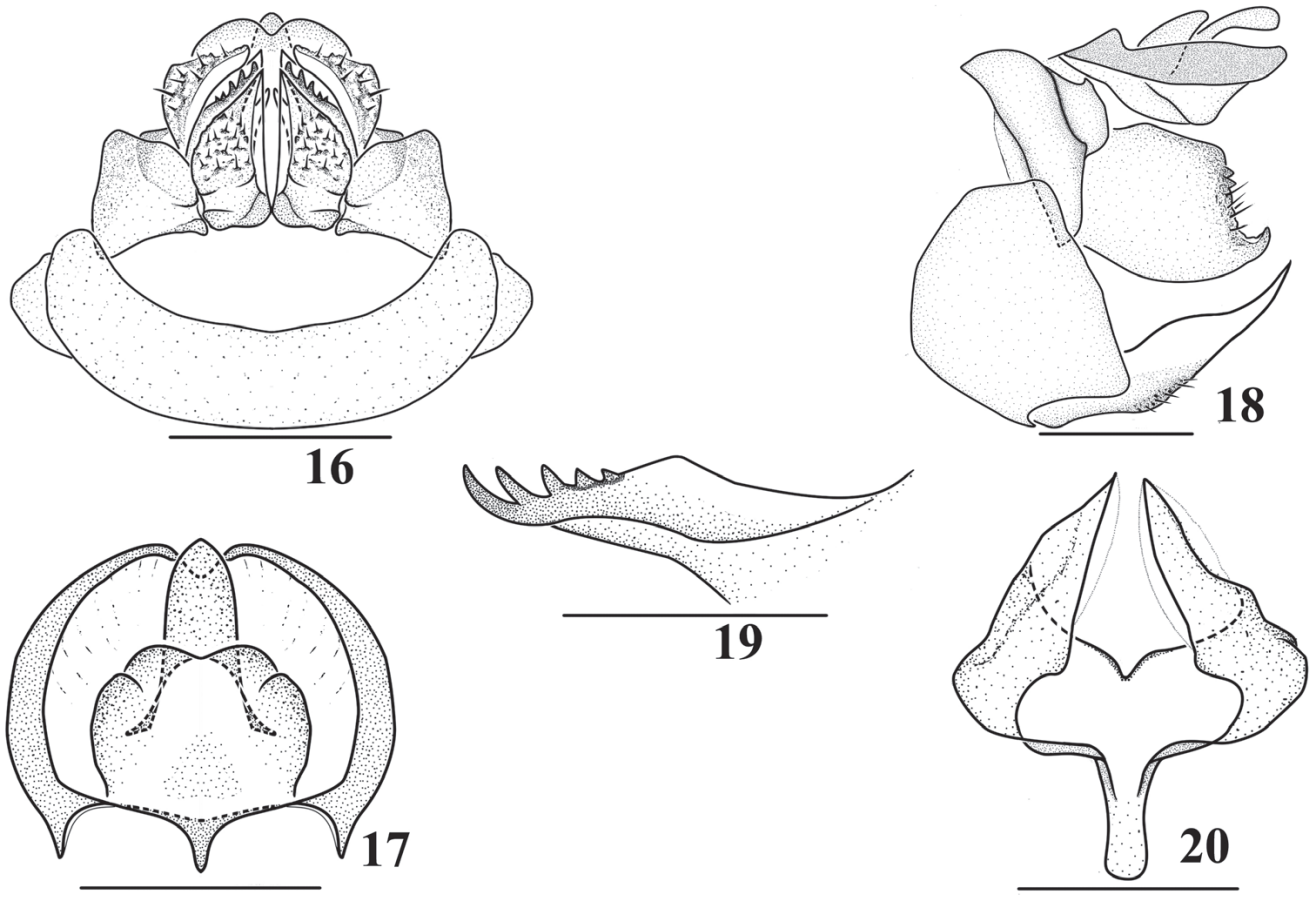
*Magadhaideus xiphos* sp. n. **16** Female genitalia, ventral view **17** Anal segment of female, dorsal view **18** Female genitalia, lateral view **19** First valvula, from inside **20** Second valvula, ventral view. Scale bars: 0.2 mm (**17–20**); 0.5 mm (**16**).

*Female genitalia.* Seventh abdominal sternum with anterior and posterior margins parallel, posterior margin truncate or slightly concave (Fig. 16). Anal segment (Figs 17–18) in dorsal view suborbicular, apical margin incised in the middle, basal margin M-shaped approximatively, with finger-like process in the middle; apex of anal stylet reaching or slightly exceeding apex of anal segment. First valvula with five spines (Fig. 19). Second valvula with two lateral lobes incompletely symmetrical, narrowing and sharp apically, directed postero-ventrally (Fig. 20). Third valvula with outer surface shagreen (Figs 16, 18); in lateral view (Fig. 18) apical margin sinuate, with an angulate process ventrally, directed inwards.

#### Remarks.

This new species differs from *Magadhaideus cervina* (Fennah, 1956) comb. n. by: forewing with a dark brown stripe from base to apex of clavus (without stripe in *cervina*); medioventral process of pygofer with two small lateroapical processes, directed outward (directed inward in *cervina*); genital style with dorsal process almost not branched into lobes (distinctly branched into three lobes in *cervina*).

#### Etymology.

The species name refers to the phallic appendage xiphoid.

#### Host plant.

Unknown.

#### Distribution.

China (Fujian, Shanxi, Jiangxi, Zhejiang, Guizhou and Guangdong).

## Discussion

On the basis of the characteristics of the vertex being at least two-thirds as wide as the pronotum and the post-tibiae with one spine characteristically present, *Magadhaideus* gen. n. is attributed to the tribe Plectoderini, following the tribal definition of Emeljanov (1992). On the basis of the peculiar characteristic of the mesonotum with a transverse callus on the anterior third of the disc, *Magadha* is clearly distinguished from other genera of Plectoderini, following the generic definition of Fennah (1950). Although the new genus also has the transverse callus, its male genitalia distinctly differs from that of *Magadha*. According to the descriptions and illustrations of *Magadha cervina* Fennah, 1956 (Fennah 1956: fig. 15: A–E), it is here attributed to the new genus.

**Figure 21.**
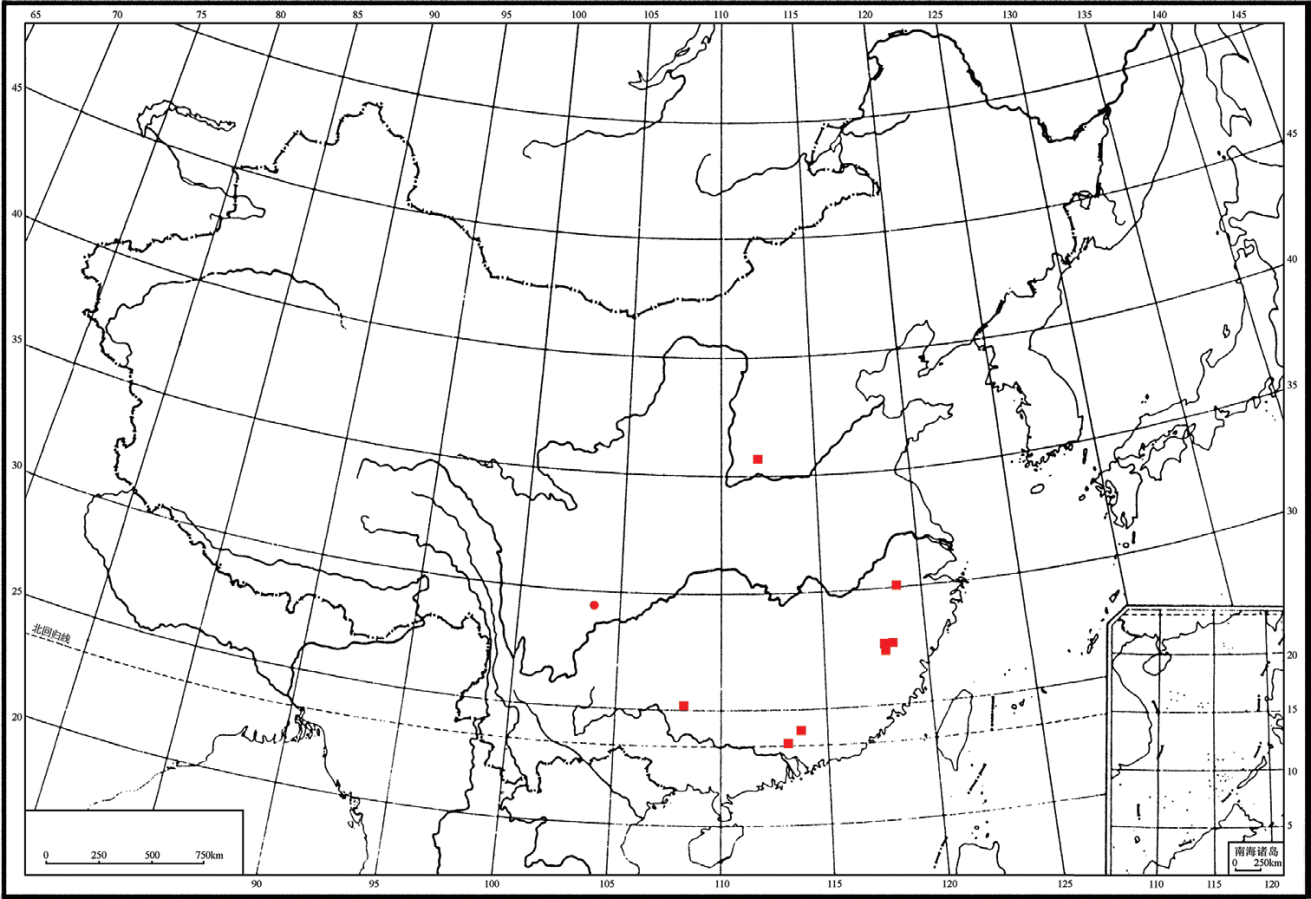
Geographic distribution of *Magadhaideus* species in China. *M. cervina* (Fennah, 1956), comb. n. (**●**); *M. xiphos* sp. n. (**■**).

The members of Plectoderini are found in seven zoogeographic regions of the world (Bourgoin 2017). Here, the new genus (Fig. 21) is distributed in the Oriental region of southern China. The adults of Plectoderini feed on the sap of trees and shrubs and the nymphs on fungi (O’ Brien 1971). However, more precise ecological records for most members of the tribe, including the hosts for *Magadhaideus* gen. n., have not yet been documented.

## Supplementary Material

XML Treatment for Magadhaideus

XML Treatment for Magadhaideus cervina

XML Treatment for Magadhaideus xiphos
